# Lead Exposure Induces Telomere Instability in Human Cells

**DOI:** 10.1371/journal.pone.0067501

**Published:** 2013-06-26

**Authors:** Géraldine Pottier, Muriel Viau, Michelle Ricoul, Grace Shim, Marion Bellamy, Corina Cuceu, William M. Hempel, Laure Sabatier

**Affiliations:** Commissariat à l’Energie Atomique (CEA), Laboratoire de Radiobiologie et Oncologie (LRO), Fontenay-aux-Roses, France; Tulane University Health Sciences Center, United States of America

## Abstract

Lead (Pb) is an important environmental contaminant due to its widespread use over many centuries. While it affects primarily every organ system of the body, the most pernicious effects of Pb are on the central nervous system leading to cognitive and behavioral modification. Despite decades of research, the mechanisms responsible for Pb toxicity remain poorly understood. Recent work has suggested that Pb exposure may have consequences on chromosomal integrity as it was shown that Pb exposure leads to the generation of γH2Ax foci, a well-established biomarker for DNA double stranded break (DSB formation). As the chromosomal localization of γH2Ax foci plays an important role in determining the molecular mechanism responsible for their formation, we examined the localization of Pb-induced foci with respect to telomeres. Indeed, short or dysfunctional telomeres (uncapped or damaged telomeres) may be recognized as DSB by the DNA repair machinery, leading to “telomere-Induced Foci” (TIFs). In the current study, we show that while Pb exposure did not increase intra-chromosomal foci, it significantly induced TIFs, leading in some cases, to chromosomal abnormalities including telomere loss. The evidence suggests that these chromosomal abnormalities are likely due to perturbation of telomere replication, in particular on the lagging DNA strand. We propose a mechanism by which Pb exposure leads to the loss of telomere maintenance. As numerous studies have demonstrated a role for telomere maintenance in brain development and tissue homeostasis, our results suggest a possible mechanism for lead-induced neurotoxicity.

## Introduction

Lead (Pb) is a naturally occurring metal which has been used for over 8000 years in a wide range of applications [1]. As Pb is non-biodegradable, it has historically been an important environmental pollutant in air and water, and while environmental Pb levels have decreased in western countries, due to strict regulatory policies, it remains a problem in developing countries, and inner city neighborhoods.

The first accurate description of symptoms from Pb poisoning was probably provided by Nicander, a little known poet of the 2nd century BC [2]. Since then, numerous studies have demonstrated that while Pb affects essentially every organ system in the body, including the hematopoietic, cardiovascular, renal and skeletal systems, it is the central nervous system which is particularly sensitive to the effects of Pb [3]. Alterations in cognitive function and behavior due to the neurotoxic effects following Pb poisoning have long been recognized [4]. Animal studies have shown that chronic lead exposure indeed affects neuronal development and adult neurogenesis [5]. Importantly, a lower threshold for the effects of Pb on the nervous system has not been determined [6]. Persistent low-level environmental contamination of this heavy metal has ensured that Pb contamination remains a significant public health issue. Lead has also been identified as a probable carcinogen [7].

Despite the fact that Pb exposure has been the subject of intense research over many decades, the mechanisms responsible for its far reaching and persistent neurotoxic effects are still poorly understood. In humans, as in animal models, Pb contamination is associated with generalized oxidative stress observed through biomarkers such as GSH status, lipid peroxidation, and an increase in the activity of glutathione reductase and peroxidase, catalase, superoxide dismutase… (reviewed in [8]). Lead serves no biological function in humans but its high electronegativity favors interactions with proteins, notably those with metal binding sites (rich in oxygen and sulfur).

More recently, the genotoxic effects of lead exposure have also been the subject of a number of studies. Using a variety of different experimental models, conditions, and endpoints, these studies have generated largely contradictory findings [9]. Nonetheless, a recently published study has reported the emergence of γH2Ax foci, commonly correlated with the formation of double-stranded DNA breaks (DSB), some hours after cell exposure to Pb [10]. Furthermore, the same study provides evidence suggesting close association between Pb molecules and DNA in the cells, suggesting that Pb directly affects DNA/chromosomal integrity.

The apparition of γH2Ax foci may arise from different mechanisms depending on the chromosomal localization. γH2Ax foci in intra-chromosomal regions are generally due to the presence of DSBs [11], whereas those at or near telomeres may also be due to telomere shortening, which has been shown to lead to γH2Ax foci formation in the absence of bona fide DSBs [12]. Telomeres are DNA-protein complexes containing long 6 base pair repeats added onto the ends of chromosomes by the enzyme telomerase. Telomeres serve multiple functions including protecting the ends of chromosomes and preventing chromosome fusion. Telomeres are maintained in germline cells, but shorten with each progressive cell division in somatic cells. Telomere shortening in somatic cells is a signal of replicative cell senescence which results from the inability of the telomere to form a cap that protects the end of the chromosome. When this occurs, the cells cease to divide and often undergo apoptosis. However, if the checkpoint pathways are deficient, cells continue to divide until they reach the stage of telomeric crisis which often leads to cell death or significant genomic instability (reviewed in [13]. When several chromosomes contain very short telomeres, their extremities tend to undergo end-to-end fusion leading to the generation of dicentric chromosomes. A correlation between reduced telomere length and dicentric chromosome frequency has been demonstrated in human senescent fibroblasts [14]. In the segregation phase of mitosis, dicentric chromosomes often form an anaphase bridge that results in chromosome breakage, increased chromosomal rearrangements and chromosomal abnormalities [15].

This association between Pb exposure and the generation of γH2Ax foci led us to study the cytogenetic consequences related to telomere maintenance. Indeed, short or dysfunctional telomeres (uncapped or damaged telomeres) may be recognized as DSB by the DNA repair machinery, leading to “telomere-Induced Foci” (TIFs). In the current study, we show that while Pb exposure did not increase intra-chromosomal foci, it significantly induced TIFs leading in some cases to chromosomal abnormalities including telomere loss. The evidence suggests that these chromosomal abnormalities are likely due to perturbation of telomere replication, in particular on the lagging DNA strand.

## Materials and Methods

### Cell Culture

Clone B3 was isolated from the human EJ30 bladder cell carcinoma cell line (obtained from Dr. William Dewey, University of California, San Francisco). The origin, derivation and use of this cell line are described in [15], [43]–[49]. Cells were grown in alpha-MEM medium (Gibco®), supplemented with 10% fetal calf serum (Eurobio) and antibiotic antimycotic (Gibco®). For the detection of γH2Ax foci in interphase nuclei, cells were seeded at 6×10^4^/slide, cultured for 24 h in the absence of Pb and a further 24 h in the presence of the indicated concentrations of Pb(NO_3_)_2_ before processing for immunofluorescence. For the detection of γH2Ax foci and/or telomeres in metaphase spreads, cells were seeded at 5×10^5^ cells/T25 flask, cultured for 24 h in the absence of Pb and a further 24 h in the presence of the indicated concentrations of Pb(NO_3_)_2_. Colchecine (6 µg/mL) was included during the last 5 hours of culture to accumulate mitotic cells before processing for FISH or IF FISH.

### Preparation of Pb Solution

Lead nitrate (Sigma Chemical) solution was prepared by dissolving 3.31 g Pb nitrate in 100 ml of distilled water (i.e. 100 mM solution).

### Chromosome Analysis

Preparation of metaphase chromosome for *in situ* hybridization was performed as previously described [50]. Cytogenetic analyses were performed in two steps, consisting of hybridization with a protein nucleic acid (PNA) telomere probe followed by M-FISH for all chromosome painting. Telomere analysis was performed as previously described [51] using telomere specific PNA probes labelled with CY3 (Perspective Biosystems). M-FISH was performed using multi-FISH probes (Metasystems Gmbh, Althusseim, Germany), according to the manufacturer’s recommendations. Images of hybridized metaphases were captured with a charge-coupled device camera (Zeiss, Thornwood, NY) coupled to a Zeiss Axioplan microscope and processed using ISIS software (Metasystems). In all cases, 50 metaphases were analysed per experiment.

### Co-FISH

Chromosome Orientation FISH (CO-FISH) consists of a FISH technique that uses single-stranded DNA probes to produce strand-specific hybridization and was performed as previously described [18]. The technique relies on labelling by 5′-bromodeoxyuridine (BrdU) (Sigma) incorporation on one strand of DNA during S-phase. Metaphase chromosomes were prepared 24 h (1 cell cycle) after the addition of 1×10^−5^ M BrdU. After rinsing the slides with SSC (Saline Sodium Citrate), they were incubated for 15 min in 5 µg/ml Hoechst 33258 (Sigma) to allow intercolation of the dye into the newly synthesized DNA. The slides were then subjected to 45 min of UV irradiation, in a bath of 2×SSC, to introduce nicks at the sites of BrdU incorporation. The nicks were enlarged by incubating the slides in 10 U/µl ExoIII (Promega) for 15 min at 37°C, leaving the parental strand as a single-stranded template for the hybridization procedure. After dehydration in successive baths of μ50, 70, and 100% ethanol for 5 min each, on ice, the slides were incubated for 1 h30 with the PNA probe T_2_AG_3_ (FITC, Fluorescein IsoThioCyanate) and then with the probe C_3_TA_2_ (Cy3) for another 1 h30. After hybridization, the slides were counterstained with DAPI (1 µg/ml).

### Immunofluorescence

Cells were fixed with methanol/acetone (1 V/1 V) for 10 min and permeabilized in 20 mM HEPES pH 7.9, 50 mM NaCl, 3 mM MgCl_2_, 300 mM sucrose 0.5% TRITON X-100 (Sigma) for 30 min. Cells were then incubated 1 h at 37°C in PBS-TRITON 0.5% with anti- γH2Ax ^ser139^ antibody (1/800; Upstate). After washing in PBS, cells were incubated with an anti-mouse CY3 secondary antibody (1/300) at 37°C for 45 min. Cells were mounted in p-phenylene diamine after counterstaining with 4,6-diamidino-2-phenylindole (Sigma).

### IF-FISH

Immunofluorescent-FISH (IF-FISH) was performed using a protocol similar to one described previously [17]. Cells were cytospun at 112×g after 5 h of colchicine (0.09 µg/ml) treatment at 37°C in a humidified atmosphere of 5% CO2. The pellet was washed in 1×PBS at 37°C and re-centrifuged. The cells were subjected to hypertonic shock by resuspension in 34 mM citrate at 37°C to obtain a suspension of cells at a concentration of 6×10^5^ cells/ml and incubated for 1 h at 37°C. 200 µl of the suspension was subsequently applied to polylysine slides by centrifugation. Following fixation (PFA 3%, sucrose 2%), cells were immunostained as already described. Prior to telomere hybridization with the PNA probe (CCCTAA)_3_- FITC, cells were successively fixed (PFA 4%, 2 min), washed in PBS and dehydrated (50/70/100 ethanol).

### Statistical Analysis

Statistical analyses of the results were performed using the student t-test for two unpaired independent sample sets (treated and untreated cells), and the standard error was calculated for chromosomal alteration frequencies between the two sample sets. The formula used to compare the two averages is shown below where s = the common variance to both sample sets, N = the number of samples.
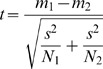



## Results

### Lead Induces γH2Ax foci that are Mainly Localized in Telomere Regions

**Figure 1:**
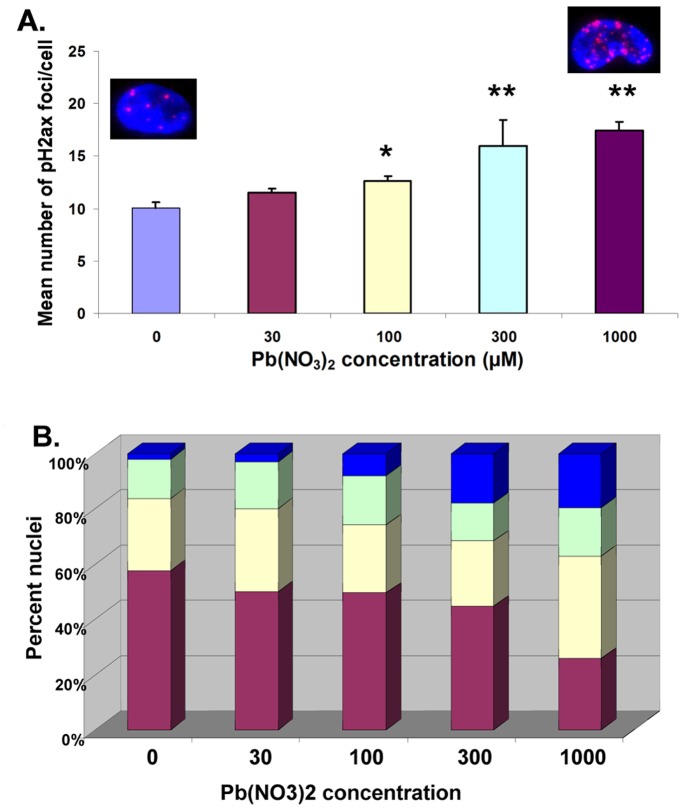
Lead induces γH2Ax foci in B3 cells. Results obtained with B3 cells following 24 h exposure with the indicated Pb(NO_3_)_2_ concentrations. **A.** Number of γH2Ax foci per cell. Inserts show representative γH2Ax signals observed for 0 and 1 mM Pb(NO_3_)_2_. **B.** Distribution of the number of γH2Ax foci per nucleus as a function of Pb concentration. Violet, 0–9 foci/nucleus; Yellow, 10–19 foci/nucleus; Green, 20–29 foci/nucleus; Blue, >30 foci/nucleus.

The induction of DNA damage following Pb nitrate exposure has been previously described, based on the quantification of nuclear foci formed following H2Ax phosphorylation by immunofluorescence (IF) [10]. They deduced that Pb induces DSB that they called HEMI-breaks for Heavy Metal Induced breaks that reached a maximum level after 24 hours of treatment. Indeed, the number of nuclear foci has been previously shown to directly correlate in a one to one ratio with the number of DSB following irradiation [16]. As γH2Ax foci formation reached a maximum level after 24 hours of Pb exposure in the paper published by Gestaldo et al, we chose to focus on the same time point for the current study. Our results confirm the generation of γH2Ax foci in human B3 cells following exposure to increasing concentrations of Pb nitrate after 24 h of treatment (**Figure 1A**). A dose dependent increase of the mean number of γH2Ax foci was observed that achieved significance starting with exposure of the cells to 100 µM Pb compared to non-exposed cells (p<0.05). Interestingly, there was a heterogeneous distribution of γH2Ax foci amongst the nuclei of B3 cells (**Figure 1B**). In untreated cells, more than 50% of nuclei contained 0–9 foci, whereas only a very small percentage contained over 30 foci. Following lead exposure, however, there was a significant increase in the percentage of nuclei containing over 30 foci starting from 100 µM (p = .0.004). Based on these results, subsequent experiments were performed with the lowest effective Pb nitrate concentration (100 µM) in order to study the mechanism and the downstream consequences of γH2Ax foci formation.

**Figure 2:**
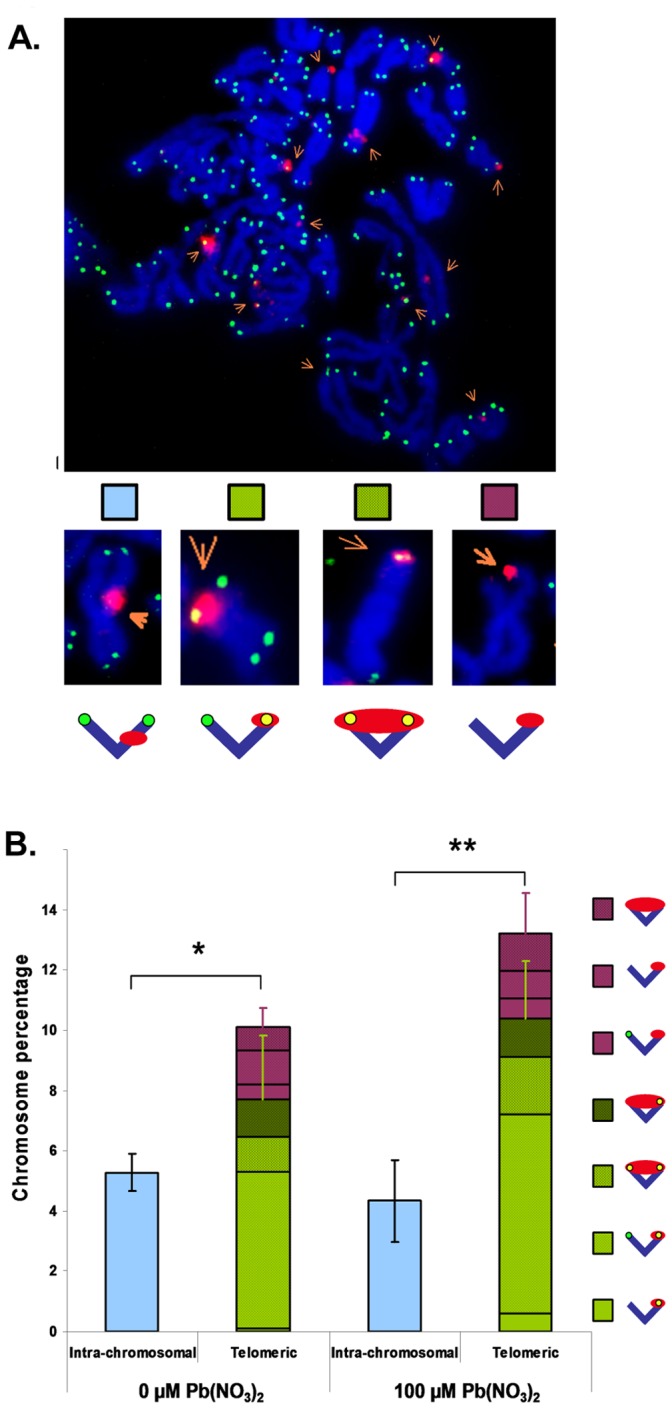
Lead induces Telomere-Induced Foci (TIFs). Results obtained with B3 cells following 24 h exposure with the indicated Pb(NO_3_)_2_ concentrations. **A.** Representative images obtained following IF-FISH. TIFs (yellow) represent co-localisation of γH2Ax (red) and telomere (green) signals. The upper panel shows a representative image of a single metaphase. The lower panel shows representative images of each type of staining pattern obtained from several metaphases **B.** Localisation of γH2Ax and telomere staining using IF-FISH. *p<0.05, ** *p*<0.01. The different combinations of staining are shown in the legend on the right.

In order to position the induced γH2Ax foci on the chromosomes, we adapted the Immunofluorescence (IF)-FISH method [17] allowing simultaneous telomere hybridization and immunofluorescent staining of γH2Ax on the same metaphase chromosomes (**Figure 2A**). Using this approach it is possible to co-localize telomeres and chromosomal γH2Ax foci, distinguish internal from telomeric γH2Ax foci, and observe telomere loss. Several examples of the chromosomal localisation of the γH2Ax foci (telomeric or intra-chromosomal) after 24 h exposure with Pb nitrate are shown in **Figure 2A**, and the comprehensive data is represented in **Figure 2B**. Several staining patterns were observed including intra-chromosomal foci or telomeric foci on one or both chromosomal arms with or without telomere staining (see next section). Surprisingly, Pb nitrate treatment (100 µM) did not impact the percentage of intra-chromosomal foci, while the percentage of telomeric γH2Ax foci (co-localized γH2Ax and telomere staining) was increased. It is important to note that the co-localisation of telomere staining and γH2Ax foci indicate that γH2Ax formation has occurred in the absence of DSBs, as if this was the case, the telomere would most likely be lost. These results strongly suggest that Pb nitrate preferentially impacts the telomeric region, leading to telomere dysfunction signalled initially as a DSB in the intact telomere.

### Lead Induces Telomere Instability

**Figure 3:**
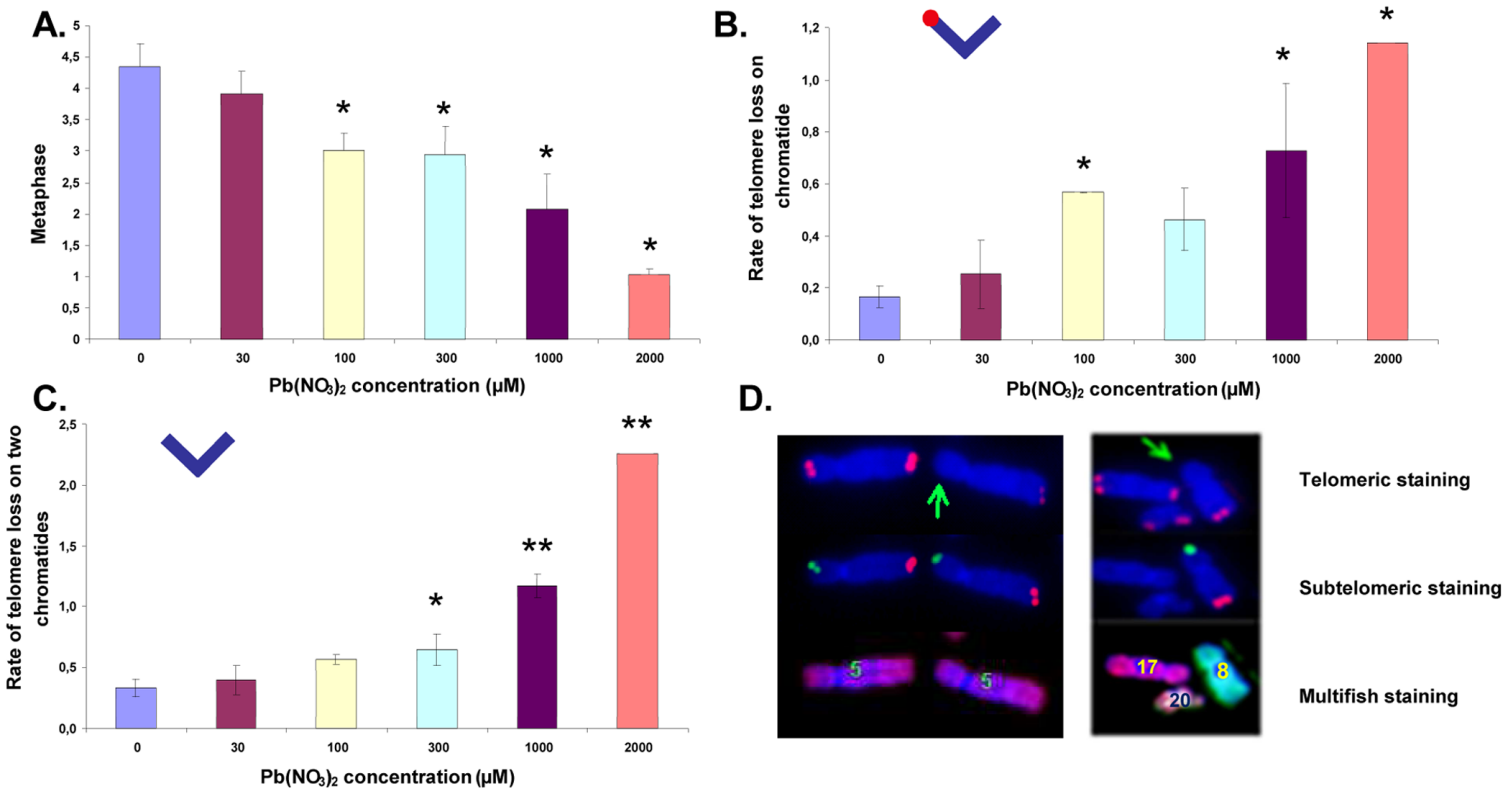
Lead induces telomere instability. **A–C.** Mean telomere loss observed in B3 cells after 24 h exposure with the indicated Pb(NO_3_)_2_ concentrations and normalized to the correspondent mitotic index. **A.** Mitotic index. **B.** Loss of one telomere on one chromatid. **C.** Loss of two telomeres on two chromatids. **D.** Representative images obtained with B3 cells following the indicated fluorescent *in situ* hybridization procedure. Subtelomere markers are: p-arm (FITC), q-arm (Texas Red).

The results obtained from the above study suggest, that in some cases, Pb exposure of B3 cells leads to telomere loss. Indeed, the preferential localization of γH2Ax foci on telomeres may indicate the presence of extremely short or dysfunctional telomeres (uncapped or damaged telomeres) that are recognized as DSB by the DNA repair machinery [12], leading to “telomere-Induced Foci” (TIFs). As Pb is cytotoxic, it was necessary to first calculate the mitotic index of cells in the presence of the different concentrations of Pb (**Figure 3A**). Pb nitrate decreased the mitotic index of treated cells. The effect was dose-dependent and statistically significant starting with exposure of the cells to 100 µM compared to non-exposed cells (*p*<0.05). These data were thereafter taken into consideration and the subsequent experiments were normalized for the decrease of the mitotic index by dividing the percentage of chromosomes exhibiting telomere loss by the mitotic index for each concentration of Pb. The telomeric status of the cells was assessed after 24 h exposure with the same concentrations of Pb nitrate. The rates of telomere loss are presented in **Figure 3B–C**. Lead exposure induced a dose-dependent increase of telomere loss which was significant from 100 µM for one chromatid. Based on these analyses, it is not possible to determine if the loss of signal for two telomeres is due to a break in the subtelomeric region or intra-chromosomally. There was also an increase in telomere doublet formation which was maximal at 100 µM but not dose dependent (see **Figure S1**). In preliminary studies, Pb induced telomere loss and doublet formation were also observed in fresh human lymphocytes (see **Figure S2**) demonstrating that Pb exposure also leads to telomere dysfunction in primary cells. Interestingly, the level of spontaneous telomere loss in, and doublet formation and the subsequent effects of Pb exposure on human lymphocytes were highly dependent on the specific donor. The mechanism behind the donor-specific variability for the effects of Pb on telomere stability is currently under investigation.

In order to determine if the observed losses were limited to the telomeric region we performed sub-telomere staining (**Figure 3D**, middle panels) on the same slides that were used for the previous study (**Figure 3D**, top panels). Unlike the telomere probe, the sub-telomere probes are chromosome-specific, necessitating chromosome identification using the multi-FISH method prior to sub-telomere staining (**Figure 3D**, lower panels). To aid in the analysis, we decided to focus on chromosomes which demonstrated an elevated frequency of telomere loss. In addition, sub-telomere probes were not available for both arms of all chromosomes, further limiting the choice of chromosomes for analysis. Based on these constraints, we selected chromosomes 3, 5, 8 and X. Representative images in **Figure 3D** show that sub-telomere staining was preserved for chromosome 5 (left panels) and chromosome 8 (right panels). These results demonstrate that the observed loss of chromosome ends following Pb nitrate treatment is strictly limited to the telomeric region as subtelomeres are the most distal regions of unique DNA sequence found on chromosomes.

### Lead Preferentially Targets the Lagging Strand

**Figure 4:**
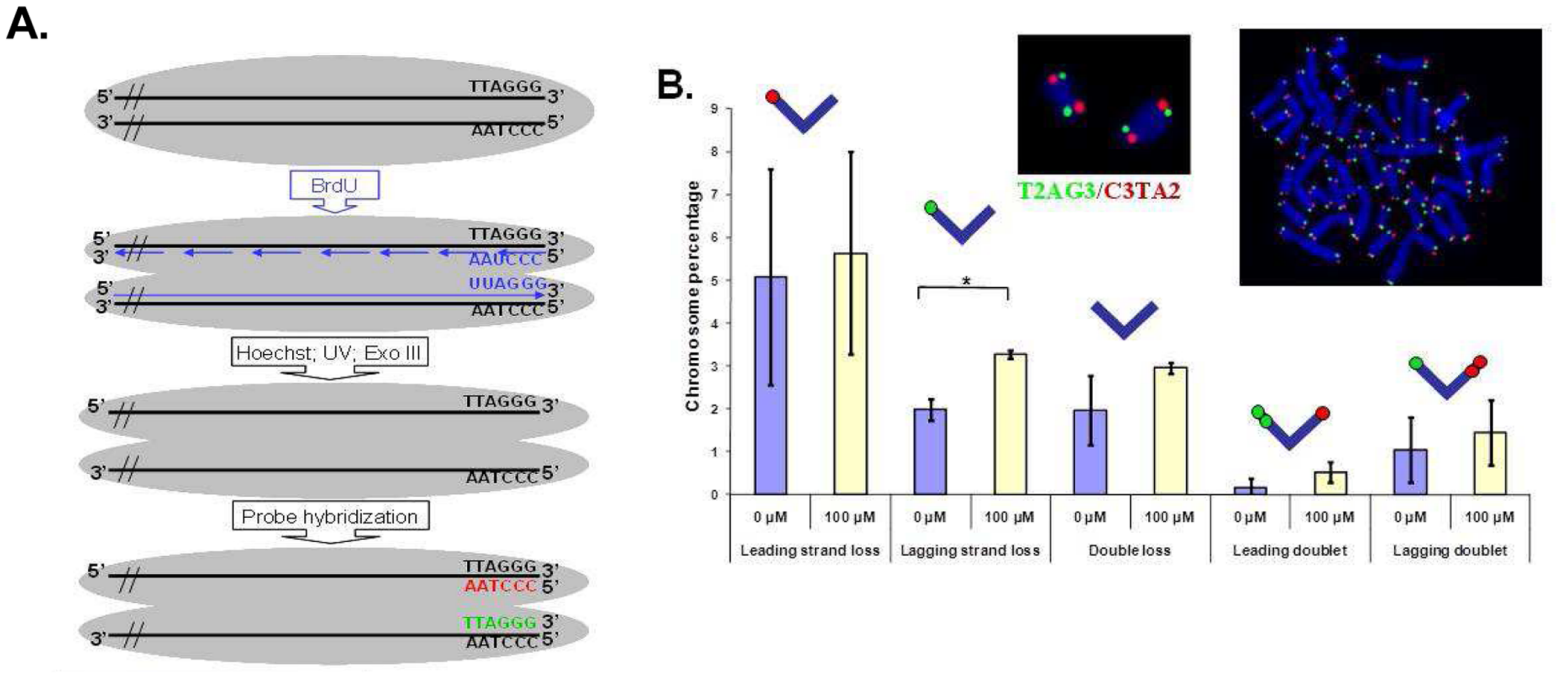
Lead induces mainly lagging strand instability. **A.** Principle of telomere hybridization by CO-FISH. **B.** Mean telomere instability observed with B3 cells after 24 h exposure with indicated Pb(NO_3_)_2_ concentrations. *p<0.05. Representative images obtained with B3 cells are shown in the inset.

As the leading and lagging strands of telomeres have dissimilar structures (reviewed in [13]), it seemed plausible that Pb may preferentially target one strand over the other. The telomeric probe used in the experiments described up to this point consisted of protein nucleic acid (PNA) bearing the specific sequence of one telomere strand. Chromosome orientation-FISH (CO-FISH) was used to differentiate the chromosomal strands [18] (lagging and leading) by the detection of their specific sequence (T_2_AG_3_ and C_3_TA_2_ respectively) with the complementary sequence (red and green respectively in **Figure 4A**). We analyzed the involvement of the leading and lagging strands in the loss of telomeres and the formation of doublets (**Figure 4B**). The presence of Pb nitrate in the cell culture medium induced a slight increase in all of the telomere aberrations analyzed. However, the increase was only statistically significant for the loss of the lagging strand (*p*<0.05). It is worth noting that FITC (green probe) is less efficient than Cy3 (red probe), and thus the loss of staining of the leading strand may be partially due to poor telomere staining, whereas the loss of staining of the lagging strand is more significant as it is likely to be a true measure of telomere loss. Interestingly, in the case of telomere doublet formation, neither strand seemed to be preferentially affected suggesting that the mechanism of doublet formation is strand-independent. These results show that exposure to Pb nitrate perturbs telomere stability and the lagging strand seems to be the preferential target of Pb nitrate induced telomere loss.

## Discussion

Despite being the subject of vigorous research, the mechanism of Pb toxicity and/or carcinogenicity remains unknown. A recent study demonstrated the appearance of γH2Ax foci, a known biomarker for DSB formation, reached a maximum 24 hours after Pb contamination [10]. In the present study we have applied cytogenetic approaches to investigate the consequences of Pb-induced γH2Ax foci formation on human B3 cells.

The mechanism behind the formation of γH2Ax foci may be quite different depending on their chromosomal localization. Intra-chromosomal foci are primarily due to DSB whereas those found at telomeres may be due to short or dysfunctional telomeres, such as those which are uncapped [19] or damaged [20]. Our results indicate that the Pb-induced γH2Ax foci form mostly at the end of chromosomes at or near the telomeres suggesting that Pb exposure may induce telomere instability most likely due to a defect in telomere maintenance resulting in the emergence of doublets and telomere loss. Sub-telomere staining confirmed that the loss is strictly limited to telomeric sequences and the Co-FISH method allowed the identification of the lagging strand as the preferential target of these losses.

Indeed, in the previous study demonstrating the appearance of γH2Ax foci following Pb administration [10], it was never conclusively proven that Pb exposure directly led to the formation of DSB despite a concerted effort to do so. The authors concluded that DSB formation was not a direct consequence of lead exposure, but only occurred at a relatively late time point due to oxidative stress and the accumulation of single stranded breaks. Indeed, the relatively late appearance of γH2Ax foci would argue against the direct formation of DSBs following lead exposure, as intra-chromosomal DSB formation normally occurs relatively rapidly following, for example exposure of cells to ionizing radiation, and are almost completely repaired at 24 hours. In light of the results of the current study, it is likely that the γH2Ax foci observed by Gastaldo et al. were actually due to the presence of dysfunctional telomeres, in the absence of DSB, following Pb exposure.

In somatic cells, telomeres shorten at each cell division representing a mitotic clock of the senescence process. Telomeres are highly sensitive to oxidative stress due to less effective DNA repair than for intra-chromosomal sequences [21] and Pb is known to perturb the oxidative status of cells (reviewed in [8]) notably through its interaction with proteins [22]. The specific targeting of telomeres could thus be indirectly attributed to Pb-induced oxidative stress. The specific alteration of this chromosomal region may have drastic consequences on the formation and the long term transmission of chromosomal rearrangements via their interplay with the natural aging of the cells [21].

The preferential targeting of Pb to the lagging strand is intriguing. The particularity of this strand is the repetition of a well conserved G-rich sequence (d[T_1–3_-(T/A)-G_3–4_)_n_) which has been shown to form G-quadruplexes *in vitro* under physiological conditions. G quadruplexes are 4 stranded nucleic acid structures which can be formed by G rich sequences and can readily form in vitro and have also been demonstrated in vivo, notably associated with telomeres. Once formed, G-quadruplexes are highly stable and have to be processed by enzymes to unwind the DNA, notably to allow telomere replication. Several enzymes have been shown to have this property: BLM, WRN, DHX36, GQN1, MRE11 and FANCJ … (reviewed in [23]; [24]). Interestingly, G-quadruplex-interacting compounds, which stabilize the G-quadruplex structure, have been shown to induce the loss of telomere maintenance leading to the induction of apoptosis in normal or tumoral cells [25]–[27].

The presence of monovalent cations is an imperative requirement for G-quadruplex formation (K^+^>Na^+^>Cs^+^>Li^+^). G-quadruplexes can also be stabilized by divalent ions, sometimes more strongly than by monovalent ions because of the increase in charge. G-quadruplex structures obtained with Pb^2+^ are more compact, the hydrogen bonds are more stable than with K^+^ [28] and far fewer Pb^2+^ ions are needed to stabilize a G-rich sequence into a G-quadruplex structure.

Based on the results of the present study, it is tempting to speculate that Pb may displace the physiological ions in already formed G-quadruplexes and possibly induces the formation of additional G-quadruplex structures. In the case of DNAzymes, the replacement of K^+^ by Pb^2+^ may inhibit the enzymatic activity because of the induced conformation changes [29]. The high selectivity of many G-quadruplex-unwinding enzymes coupled with their high affinity for these structures could lead to their inability or a reduced ability to recognize and/or cleave the Pb-compacted G-quadruplexes.

Several studies have shown that cells carrying defective G-quadruplex-unwinding enzymes demonstrate telomere instability. For example, cells deficient for the Werner Syndrome RecQ Helicase (WRN), known to be critical in the processing of G quadruplexes, are characterized by the preferential loss of telomeres from the lagging strand [30]. The telomere instability observed in the present study could be correlated with the persistence of the G-quadruplexes during replication, underling a defect in telomere maintenance during replication.

The effects of lead exposure on telomere maintenance are also intriguing in light of numerous recent studies which demonstrate a role for telomere maintenance in brain development and tissue homeostasis suggesting a possible mechanism for lead-induced neurotoxicity. Studies in mice in which either the telomerase mRNA component (mTR) [31] or telomerase reverse trascriptase (mTERT) [32] have been deleted demonstrate progressively shorter telomeres leading to numerous problems in organs with high cellular turnover after 4–6 generations [33], [34]. In addition, starting from the 4^th^ generation, developing embryos exhibit a failure in neural tube closure [35]. Furthermore, telomere shortening in neural stem cells has been shown to disrupt neurogenesis [36]. Interestingly, the effects of TERT deletion can be reversed in aging mice by inducible expression of TERT, leading to an increase in telomere length, and the reversal of tissue degeneration including in the central nervous system [37]. More recently, a strong link between levels of TERT activity in the hippocampus and depression has been demonstrated in a chronic mild stress mouse model [38].

In humans, perceived chronic stress [39] and high phobic anxiety [40] have been shown to be associated with telomere shortening of blood lymphocytes suggesting a link between environmental stressors, disruption of telomere maintenance, and psychological state. Recently, an age-independent association was also demonstrated between telomere length and cognitive function in a cohort of elderly subjects [41]. It is noteworthy that similar psychological and cognitive effects have been linked to childhood Pb exposure.

Finally, a recently published study has demonstrated that occupational exposure is associated with telomere shortening in the peripheral white blood cells of Chinese battery manufacturing plant workers [42] that was directly proportional to the body lead burden, suggesting a link between occupational lead exposure and the loss of telomere maintenance. While this study only demonstrates an association between lead exposure and telomere disruption, these results take on new significance in light of the results presented here which strongly suggest a causal link between lead exposure at the cellular level and the loss of telomere maintenance.

A model for telomere instability following Pb exposure which could explain Pb-induced neurotxicity can be proposed. The greater affinity of Pb for G-rich sequences may induce the displacement of the physiological ions (K^+^ or Na^+^) to form tighter G-quadruplex complexes [29]. The complexes need to be processed to permit telomere replication but the highly specific unwinding enzymes [23] may not function properly due to the more highly compacted form of the complex. Alternative mechanisms are perhaps then recruited to obtain linear DNA, notably with the intervention of nucleases that perturb telomere integrity leading to their recognition as DSBs (γH2Ax staining). We propose that the first anomaly is the emergence of telomere doublets subsequent to processing by the alternative unwinding enzymes. The loss of telomere integrity then leads to the loss of the telomeric sequence on one or sometimes two chromatids. This hypothesis is consistent with the results observed where the rate of doublet formation is not dose-dependent and with the γH2Ax staining at telomeres while telomere sequences are still detectable. The subsequent impaired telomere maintenance induces genomic and chromosomal instability that may lead to a perturbation in neurogenesis resulting in Pb-induced neurotoxicity. The loss of telomere maintenance may also be directly neurotoxic, but this remains to be demonstrated.

In light of our current knowledge of telomeres and the critical role that they play in cellular aging as well as in the development and maintenance of the central nervous system, and the downstream repercussions for cognitive and psychological status, the results presented from the current study suggest a promising novel avenue of inquiry to better understand the link between Pb exposure, telomere maintenance, and the biological responses leading to the permanent nature of the resulting neurotoxic effects.

## Supporting Information

Figure S1 **Lead induces the appearance of telomere doublets.** Appearance of telomere doublets observed in B3 cells after 24 h exposure with the indicated Pb(NO_3_)_2_ concentrations and normalized to the corresponding mitotic index.
(TIF)

Figure S2 **Lead induces telomere instability in primary human blood lymphocytes.** To measure inter-individual variability, the spontaneous level of telomere loss and doublet formation were measured in the blood lymphocytes of 20 healthy donors (Panels A and B). The values represent the mean of the independent analyses of 3 different evaluators. The effect of Pb on telomere loss and doublet formation were measured in human blood lymphocytes of 7 donors after 24 h exposure with the indicated Pb(NO_3_)_2_ concentrations (Panels C and D). *p<0.05, ** p<0.01. In all cases, 50 metaphase spreads were analyzed.
(TIF)
