# Supplementary figures and images for: Lead Exposure Induces Telomere Instability in Human Cells
Source: PLoS One. 2013 Jun 26;8(6):e67501. doi: 10.1371/journal.pone.0067501 (PMC3694068; doi:10.1371/journal.pone.0067501)

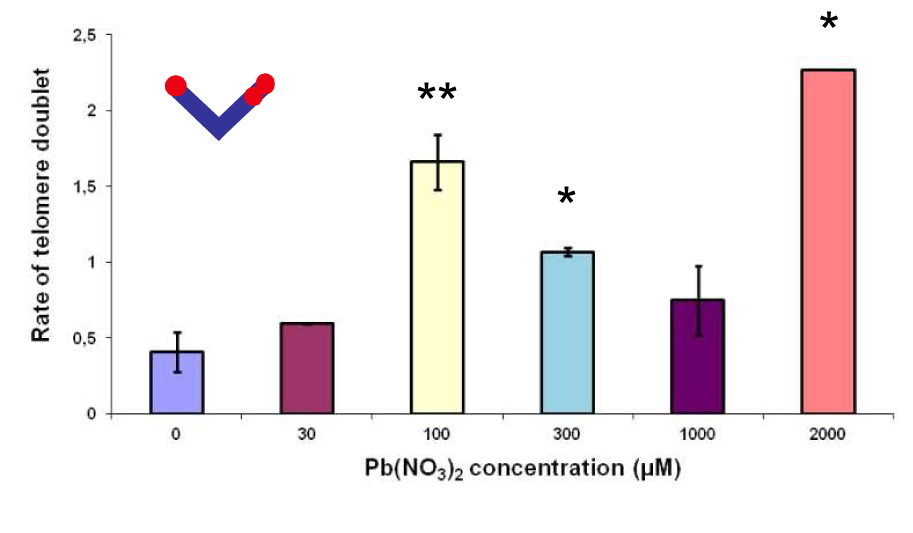

Supplement: Figure S1 — Lead induces the appearance of telomere doublets. Appearance of telomere doublets observed in B3 cells after 24 h exposure with the indicated Pb(NO3)2 concentrations and normalized to the corresponding mitotic index. (TIF) [file pone.0067501.s001.tif]

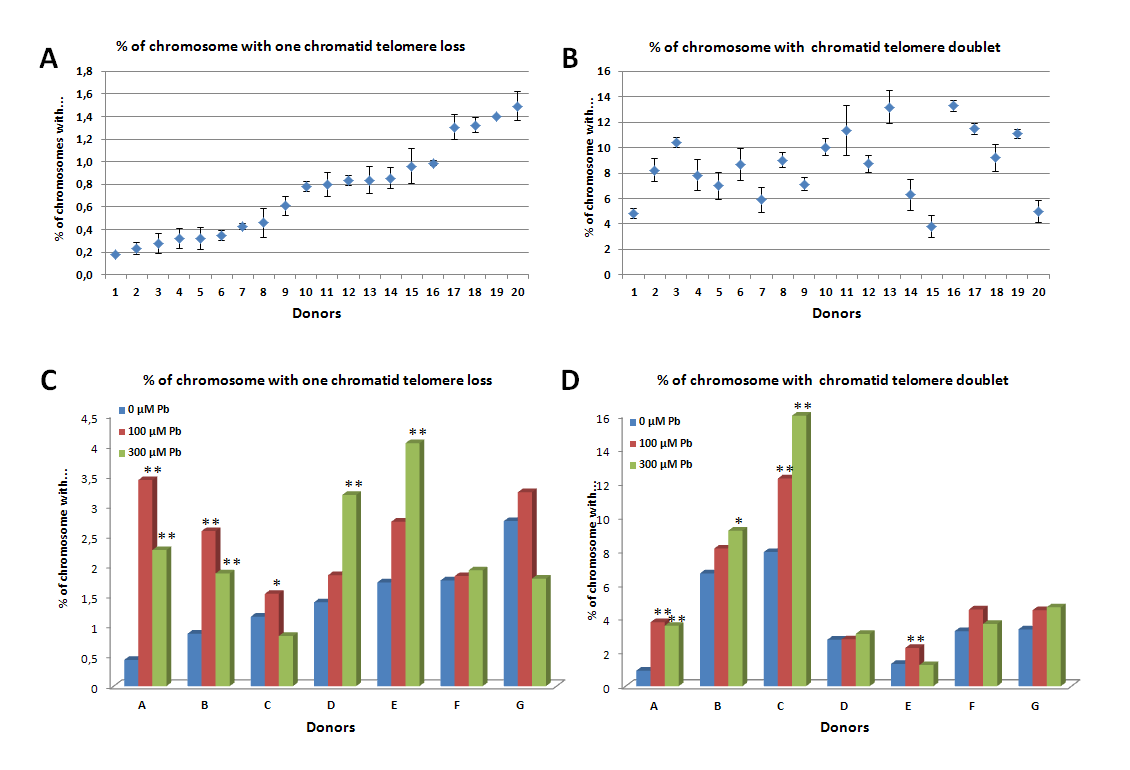

Supplement: Figure S2 — Lead induces telomere instability in primary human blood lymphocytes. To measure inter-individual variability, the spontaneous level of telomere loss and doublet formation were measured in the blood lymphocytes of 20 healthy donors (Panels A and B). The values represent the mean of the independent analyses of 3 different evaluators. The effect of Pb on telomere loss and doublet formation were measured in human blood lymphocytes of 7 donors after 24 h exposure with the indicated Pb(NO3)2 concentrations (Panels C and D). *p<0.05, ** p<0.01. In all cases, 50 metaphase spreads were analyzed. (TIF) [file pone.0067501.s002.tif]
